# Acute hyperglycemia compromises the responses of choroidal vessels using swept-source optical coherence tomography during dark and light adaptations

**DOI:** 10.3389/fendo.2023.1049326

**Published:** 2023-02-09

**Authors:** Zhiyang Lin, Huankai Yu, Ce Shi, Hongling Chen, Guangqing Lin, Meixiao Shen, Chenxiao Wang

**Affiliations:** ^1^ Eye Hospital and School of Ophthalmology and Optometry, Wenzhou Medical University, Wenzhou, China; ^2^ Oujiang Laboratory (Zhejiang Lab for Regenerative Medicine, Vision and Brain Health), Eye Hospital, Wenzhou Medical University, Wenzhou, Zhejiang, China

**Keywords:** hyperglycemia, choroid, choroidal vascularity index (CVI), swept-source optical coherence tomography (SSOCT), dark and light adaptation

## Abstract

**Purpose:**

To clarify the effects of acute hyperglycemia on the responses of choroidal structural components and vascularity index during light modulation in healthy participants using techniques including image binarization and artificial intelligence (AI) segmentation based on swept-source optical coherence tomography (SS-OCT).

**Methods:**

Twenty-four eyes of 24 healthy participants were imaged at different stages after ambient light, 40 min of dark adaptation, and 5 min of light adaptation in two imaging sessions: control and after receiving 75 g of oral glucose solution. The choroidal structural parameters, including luminal volume (LV), stromal volume (SV), total choroidal volume (TCV), and choroidal vascularity index (CVI) within a 6 mm area were determined using a custom algorithm based on image binarization and AI segmentation of SS-OCT. These measurements were compared among the conditions after adjusting for axial length, age to identify the differences.

**Results:**

In the dark, CVI decreased (-0.36 ± 0.09%) significantly in acute hyperglycemia compared to the control condition. During the transition to ambient light, there was an increasing trend in the choroidal parameters compared with the control experiment. However, only TCV (0.38 ± 0.17 mm^3^) and LV (0.27 ± 0.10 mm^3^) showed a significant increase at the time point of 5 min after ambient light.

**Conclusion:**

Analysis of choroidal structural parameters and CVI based on SS-OCT images is a potentially powerful method to objectively reflect subtle changes in neurovascular coupling between the choroid and photoreceptor during dark adaptation.

## Introduction

Rod and cone photoreceptor cells are the most prevalent cells in the retina, and their highest oxygen demand occurs in the dark (1, 2). Accumulating evidence suggests that hyperglycemia is associated with suppression of photoreceptor metabolism and increased consumption of oxygen and glucose (3). As the major blood supply system for photoreceptor cells, the vascular responses of the choroid may be compromised while modulating light adaptation and glucose. A confocal laser Doppler flowmeter study demonstrated decreased choroidal blood flow during dark adaptation in normal human eyes (4). Fuchsjäger-Mayrl et al. also found that choroidal blood flow decreased during dark adaptation and increased during light adaptation (5). Using OCT, Alagoz et al. showed that subfoveal choroidal thickness increased during dark adaptation and decreased during light adaptation (6).In addition, Kwan et al. found that hyperglycemia leads to a complete reversal of dark/light adaptation trends in the vessel density of the inner retinal capillary plexus (7). However, it is unclear whether acute hyperglycemia affects any of the choroidal changes that occur during dark adaptation and transition to light. Determining the effects of acute hyperglycemia on the choroid may improve our understanding of the mechanisms of retinal diseases, including diabetic retinopathy (DR), and help identify novel biomarkers for early diagnosis and treatment.

The advent of SS-OCT allows for more precise, non-invasive imaging of the choroid *in vivo* than SD-OCT (8, 9). The techniques used in the previous studies assess only changes in the choroidal thickness, which includes both lumina and stroma, and it is not clear whether the change is caused by LA or SA. Furthermore, it lacks repeatability, and changes in refractive errors vary significantly with age, sex, and axial length (10). Agrawal et al. first proposed the Niblack image binarization technique (10) to differentiate lumina and stroma and defined the luminal area (LA), stromal area (SA), total choriodal area (TCA), and choroidal vascularity index (CVI) as quantitative parameters to assess choroidal structures through EDI-OCT scans (10). This method has been validated with good reproducibility and also applied in research in many diseases, such as DR (11), central serous chorioretinopathy (CSC) (12), age-related macular degeneration (AMD) (13). Recently, our group developed an AI segmentation combined with Niblack’s automatic local threshold to obtain choroid volume parameters (14). Therefore, choroidal structural parameters, including luminal volume (LV), stromal volume (SV), total choroidal volume (TCV), and CVI, defined as the ratio of LV to TCV, may be potential quantitative indicators for characterizing the choroidal response while modulating light adaptation and blood glucose.

In this study, we sought to clarify the effects of acute hyperglycemia on the responses of choroidal structural components and the vascularity index during dark adaptation and transition to ambient light in healthy participants using techniques such as image binarization and AI segmentation based on SS-OCT.

## Materials and methods

Twenty-four healthy young adults were recruited from the students and work staff of theWenzhou Medical University. This prospective cross-sectional study was conducted at the Affiliated Eye Hospital of the Wenzhou Medical University. This study was conducted in accordance with the tenets of the Declaration of Helsinki and approved by the Ethics Committee of Wenzhou Medical University (Approval ID. 2022-142-K-111-01). Written informed consent was obtained from all the participants before the examination. Inclusion criteria: All the participants underwent basic ophthalmic examinations performed by experienced ophthalmologists (ZL and HY). The axial length (AL) was measured using Lenstar LS 900 (Haag Streit, Koeniz, Switzerland). BCVA was determined using subjective refraction. All participants were imaged during their working hours (from 8:00 AM to 12:00 AM), and participants with a BCVA of 20/20 or better in the right eye were recruited. The exclusion criteria were ocular diseases affecting best-corrected visual acuity (BCVA) and retinal morphology, systematic diseases, history of ocular surgery and trauma, and relevant ocular treatment.

### OCT image acquisition

**Figure 1:**
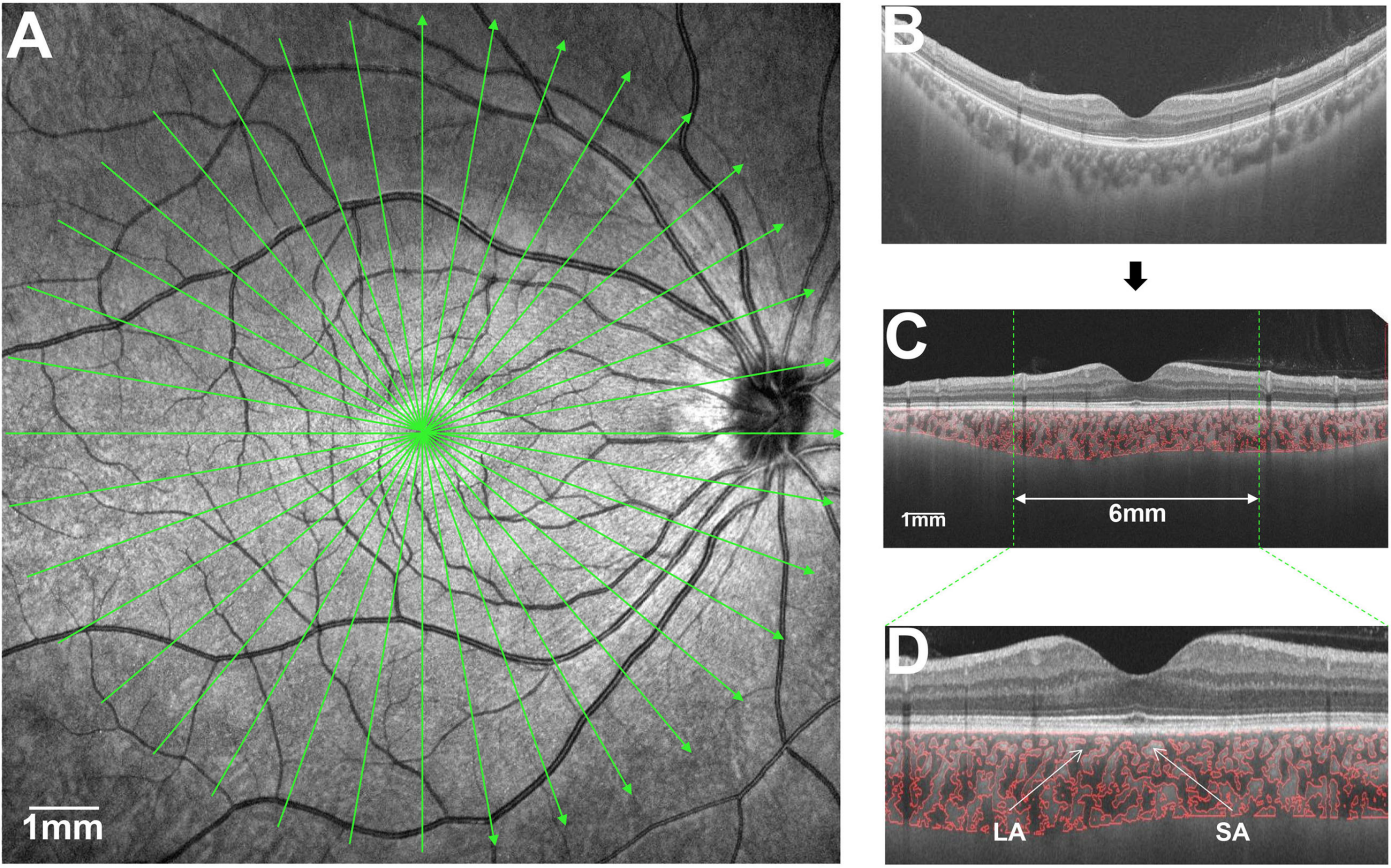
Illustration of choroidal analysis. **(A)**, scan range of choroid within 12 mm with 18 radial lines; **(B)**, Raw images of the choroid; **(C)**, Choroidal vascular and stroma image is flattened; **(D)**, Magnified Choroidal vascular and stroma imagein 6 mm.

Only the right eyes of the participants were imaged using SS-OCT (VG200 S, SVision Imaging, Henan, China), as described in detail in our previous studies (8, 9). Briefly, this SS-OCT device had a central wavelength of 1050 nm, axial resolution of 6.3 μm, lateral resolution of 13 μm, speed of 200,000 A-scans/s, and scan depth of 3 mm (8, 9). For the acquisition of SS-OCT structural images, 18 radial scan lines centered on the fovea were obtained. Each scan consisted of 2048 A-lines, had an image width of 12 mm, and was performed at intervals of 10°. Every structural scan was repeated for 16 B-scans to reduce the noise and improve the signal-to-noise ratio. Each layer of the retina and choroid boundaries could be clearly visualized in a single SS-OCT structural image. The total acquisition duration was approximately 3 seconds (**Figure 1A**).

All the images were visually checked by two experienced ophthalmologists (ZL and HY), and only images with quality scores ≥ 8 and without signs of extensive eye movement and image artifacts were selected for further image analysis.

### Imaging protocol

**Figure 2:**
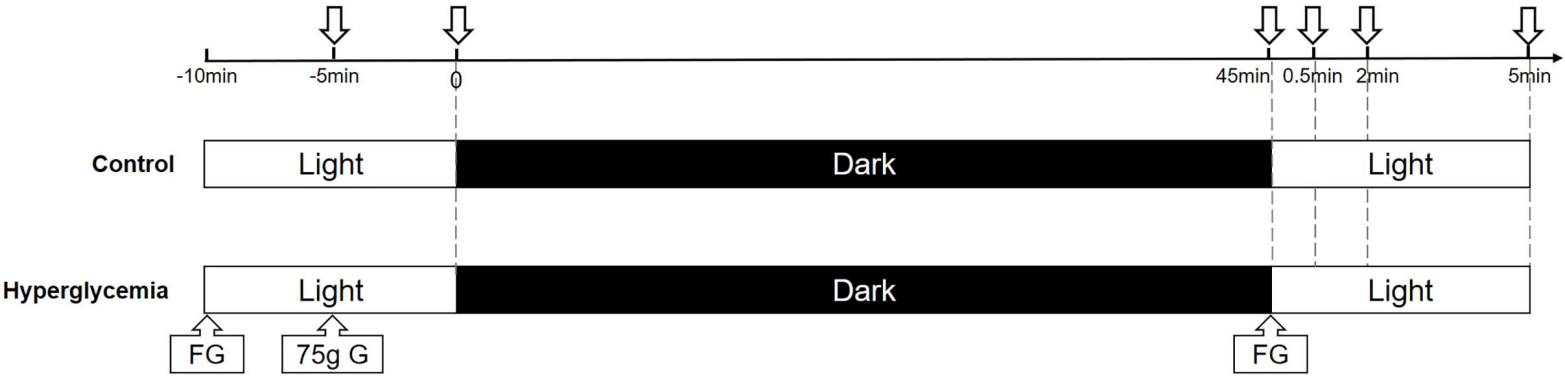
A schematic of dark and light adaptation image acquisition. Images were acquired at baseline in ambient light, after 45 min of dark adaptation, and at several time points (30 s, 2 min, 5 min) after transitioning from dark to ambient light in the control and hyperglycemia conditions. FG, finger blood sugar; G, glucose.

In the control experiment, all participants were instructed to look at a distance for 10 min before the first SS-OCT scan. All the participants underwent OCT imaging of the choroid at ambient level. Subsequently, all the participants underwent dark adaptation by staying in a completely dark room (0 cd/m^2^) and wearing a thick eye patch over the right eye for 45 min (15). Each participant was instructed to undergo OCT by moving the eye patch and scanning the choroid around the macula. During imaging, the internal fixation light in the SS-OCT system was turned off to ensure that the eye was still dark adapted (16). The room light was turned on to ambient levels (200 cd/m^2^), and participants were instructed to obtain choroid SS-OCT images at three time points (30 s, 2 min, and 5 min) (**Figure 2**).

In the glucose experiment, baseline blood glucose levels were measured at the same time the next day. Each participant was given 10 min to consume a standard 75 g oral glucose tolerance test solution (17). The participants then underwent dark adaptation and OCT imaging, as described above, and the blood glucose level was measured again after dark adaptation.

The same procedure was used in both the experiments. Before the study, all subjects were instructed not to consume any alcohol (18) or caffeinated drinks (19), refrain from performing workouts for 24 hours (20), and not to consume any food or drinks other than water for 8 hours prior to the experiment.

### Image analysis

A custom algorithm based on image binarization and AI segmentation of SS-OCT (14) was used to obtain the choroidal structural parameters and vascularity index, which has been described in detail in our previous studies (9, 21). Briefly, the upper and lower boundaries of the choroid were automatically detected using an algorithm based on the deep learning network implemented in MATLAB 2017a (Mathworks, Inc., Natick, MA, USA). Subsequently, using the Niblack’s automatic local threshold technique, the choroidal luminal area and stroma were divided (12, 22). Bennett’s formula was used to adjust the magnification of the OCT imaging system camera (23). Before the volumetric measurements of the choroid, the foveal center was manually marked by an experienced ophthalmologist (ZL), and the images were flattened by the reflection of the retinal pigment epithelium to reduce the image tilt angle, which affected the calculation of the anatomical parameters of the choroid. After the reconstruction, the LV, SV, and TCV in the range of 0 – 3 mm, 3 – 6 mm, 0 - 6 mm around the macula were separated and the CVI was derived by calculating the ratio of LV to TCV (**Figures 1B–D**).

### Statistical analysis

All statistical tests were performed using the R software (version 4.1.1). The choroidal structural parameters were compared at each time point in both the control and hyperglycemic conditions using mixed-effects linear regression models to adjust for axial length, age. The paired-samples T test was used to test the changes in choroidal structural parameters after dark adaptation and transition to ambient light. Statistical significance was set at P < 0.05. All choroidal structural parameters and the vascularity index are presented as the mean ± standard error.

## Results

Twenty-four right eyes from 24 healthy participants (9 men and 15 women) with a mean age of 25.29 ± 1.91 years were enrolled in this study. At baseline in the control condition experiment, the mean TCV was 26.08 ± 1.30 mm^3^. The mean luminal and stromal volumes were 15.34 ± 0.78 mm^3^ and 10.74 ± 0.54 mm^3^, respectively, which yielded a mean CVI of 58.77 ± 0.29% (**Table 1**). There was no significant difference in any measurement of the choroidal structural parameters and CVI between the two experiments in the control condition and hyperglycemia at baseline (all P > 0.05). Blood sugar levels increased significantly after consumption of the glucose solution (P < 0.001) in the hyperglycemia experiment. The basic information of the enrolled participants was shown in (**Table 1**).

**Table 1:** Participants’ characteristics.

Characteristic	Value
Number of females, n (%)	15 (62.5)
Age (years)	25.29 ± 1.91
SE (Diopter, D)	-4.41 ± 2.22
AL (mm)	25.47 ± 1.16
Blood glucose, mmol/L	
Pre-oral glucose tolerance test	5.03 ± 0.34
Post-oral glucose tolerance test	7.70 ± 1.48
Change	2.67 ± 1.49^*^
Choroidal parameters at baseline	Control vs. Hyperglycemia
LV (mm³)	15.34 ± 0.72 vs. 15.32 ± 0.66
SV (mm³)	10.74 ± 0.50 vs. 10.72 ± 0.46
TCV (mm³)	26.08 ± 1.21 vs. 26.04 ± 1.11
CVI (%)	58.77 ± 0.27 vs. 58.81 ± 0.28

^*^: P<0.05.

**Figure 3:**
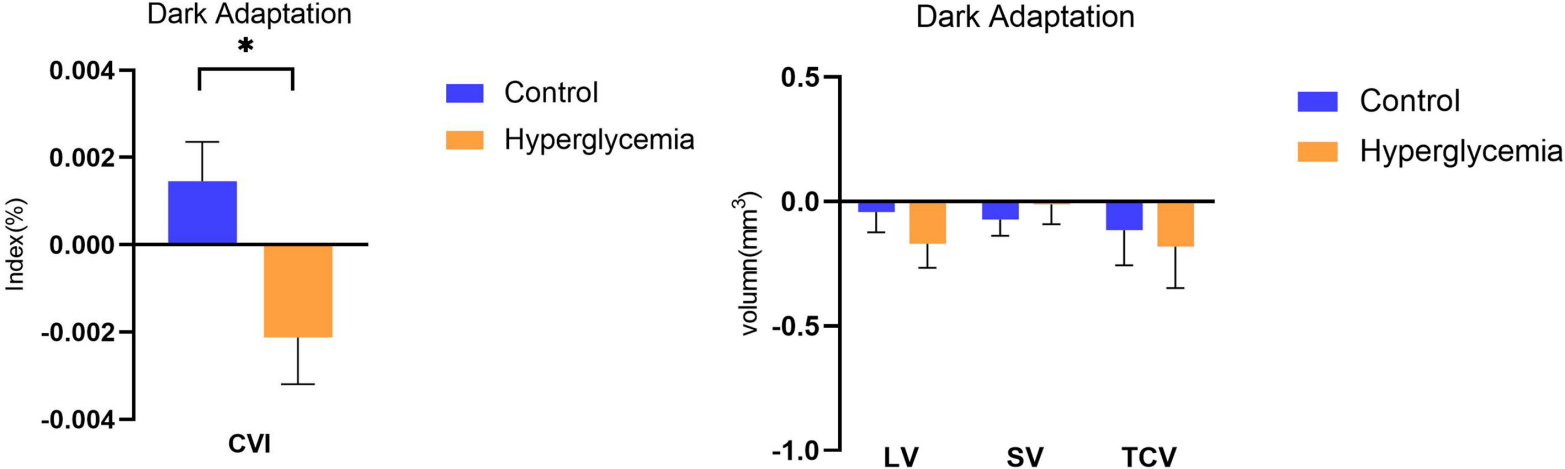
Difference in choroidal parameters between the glucose condition and control during dark adaption. Asterisks represent a significant difference between hyperglycemia and control (P < 0.05). CVI, choroidal vascular index; SV, stromal volume; TCV, total choroidal volume; LV, luminal volume.

After adjusting for axial length, age, we compared the parameters of the structural choroid in hyperglycemia to those in control conditions. After dark adaptation in the control condition, there was a decreasing trend in the choroidal structural parameters, including LV, SV, and TCV, and an increasing trend of CVI compared to the baseline; in the hyperglycemia condition, all choroidal structural parameters and CVI showed a tendency to decrease (**Figure 3**). However, only the difference in CVI from light to dark adaptation reached significance between the hyperglycemic and control conditions (P < 0.001, **Figure 3**, **Table 2**).

**Table 2:** Mean differences in choroidal parameters change between glucose and control conditions during light modulation.

		Light Adaption		
	Dark Adaption	30 secs	2 min	5 min
Parameter				
**TCV (mm³)**	-0.07 ± 0.15	0.18 ± 0.14	0.25 ± 0.17	0.38 ± 0.17^*^
**P value**	0.655	0.197	0.161	0.034
**LV (mm³)**	-0.13 ± 0.08	0.12 ± 0.08	0.20 ± 0.10	0.27 ± 0.10^*^
**P value**	0.135	0.137	0.069	0.013
**SV (mm³)**	0.06 ± 0.08	0.06 ± 0.08	0.05 ± 0.08	0.11 ± 0.08
**P value**	0.443	0.435	0.556	0.187
**CVI (%)**	-0.36 ± 0.09^*^	-0.09 ± 0.33	-0.14 ± 0.33	-0.11 ± 0.33
**P value**	<0.001	0.453	0.099	0.230

Mean difference (glucose – control) ± SE.

TCV, total choroidal volume; LV, luminal volume; SV, stromal volume; CVI, choroidal vascularity index.

^*^: P<0.05.

**Figure 4:**
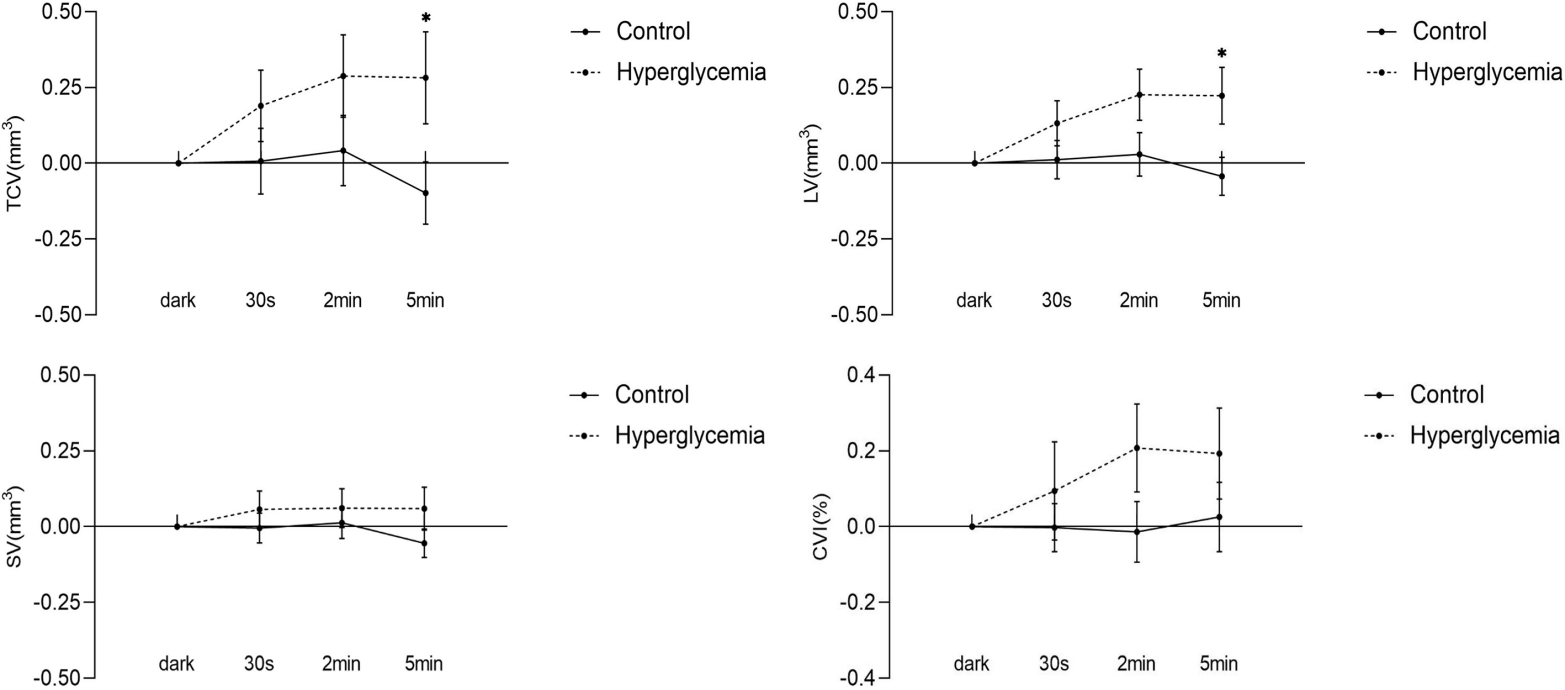
Difference in choroidal parameters between the glucose condition and control during light adaption. Asterisks represent a significant difference between hyperglycemia and control (P < 0.05). CVI, choroidal vascular index; SV, stromal volume; TCV, total choroidal volume; LV, luminal volume.

With the transition to ambient light, hyperglycemia causes an increase in LV, SV, TCV, and CVI. In the control condition, there was no significant change in any of the choroidal structural parameters and CVI, although it appeared that there was a decreasing trend in the volumes of stroma and lumina, and total choroid described by LV, SV, and TCV at the time point of 5 min of ambient light. In hyperglycemia, LV, SV, TCV, and CVI increased compared to those in the dark. However, only the differences in LV (P = 0.013, **Figure 4**) and TCV (P = 0.034, **Figure 4**) between hyperglycemia and control groups reached significance at the time point of 5 min of ambient light (**Table 2**). We found similar results in 0– 3 mm and 3 – 6 mm regions (**Supplement Tables 1**, **2**).

## Discussion

Since Agrawal et al. first proposed the Niblack image binarization technique for EDI-OCT images, the choroid can be differentiated into luminal and stromal components (10, 14). Through the acquisition of more detailed information on the choroidal structures using the deep-learning-based automatic segmentation and modified image binarization technique, our study demonstrated the effect of acute hyperglycemia on the choroidal components and CVI while modulating light adaptation in healthy participants. Under hyperglycemia in the dark, LV and TCV showed a decreasing trend, and CVI decreased compared to the control, although only CVI reached significance. This suggests that an eye with hyperglycemia was likely to exhibit a decreased range of reaction in lumina and a smaller reaction in stroma during darkness, likely underlying the observed decrease in CVI.

In darkness, the metabolic needs of the photoreceptors reach their peak (1, 2) in the absence of autoregulation of the choroidal vasculature. The photoreceptors residing in the zone where both the choroidal and retinal circulations provide oxygen support (24). F Scarinci et al. found that macular photoreceptor disruption in patients with diabetic retinopathy corresponds to areas of capillary non-perfusion at deep capillary plexus (DCP) using OCT/OCTA. This demonstrates that DCP ischemia contributes to disruption of the photoreceptor layers (25). As shown in another study (7), in the dark, there was a decrease in DCP in hyperglycemia. Previous studies had shown that photoreceptor metabolism induced physiological hypoxia in the retina (26) and this episode induced retinal glial cells to express the hypoxia-inducible vascular endothelial growth factor (VEGF) (27, 28).Furthermore, Saint-Geniez M et al. found that VEGF signaling was involved in the maintenance of the choriocapillaris, which might affect choroidal blood flow change (29).Therefore, during dark adaptation, photoreceptors were exposed to a relatively hypoxia environment due to metabolic demands. We speculated that VEGF secreted by retinal glial cells was received by choriocapillaris, resulting in increased in choroidal blood flow in normal subjects to meet the oxygen consumption of photoreceptors. Thus, the finding of significantly decreased CVI with hyperglycemia in the dark may further lead to relative photoreceptor hypoxia, which requires further validation of this concept.With transition to ambient light, LV, TCV, and CVI increased in hyperglycemia compared to the control, and only the increased LV and TCV reached a significant change. There were no significant differences in CVI between the hyperglycemia and control conditions. This suggests that hyperglycemia mainly causes vasodilatation of choroidal lumina during transition to ambient light. Several studies have shown that hyperglycemia decreases blood flow in the choroid (30, 31). Based on this evidence, we speculated that during hyperglycemia, choroidal LV increases in the face of overall decreased flow, which could indicate a reactive vasodilator response at the choroidal lumina in response to the overall decreased flow. This needs to be validated in future studies.

To the best of our knowledge, this is the first study to explore the effects of acute hyperglycemia on choroidal structural components and vascularity index while modulating light adaptation and glucose levels in healthy subjects. Current evidence suggests that acute hyperglycemia affects the response of the choroid vessels during dark/light adaptation. These findings could have important implications for DR. Changes in the underlying choroidal vasculature may contribute to the pathophysiology of DR. Choroidal vascular degeneration, choroidal aneurysms, choriocapillaris dropout, choroidal neovascularization, blood flow change, and increased tortuosity and narrowing of the vessels were evident in previous histopathological and animal studies in diabetic (32, 33). A previous study by our group demonstrated that choroidal thickness decreased in the eyes with no DR and in the eyes with mild to moderate NPDR (34). In addition to the choroidal thickness, CVI also decreases at the early stage of DR (11, 35, 36). Damian et al. showed that an increase in RPE thickness, which points to compromised function of the outer retinal layers, was associated with CVI in the eyes with DR (37). Based on these research evidences, we assume that the decreased changes in choroidal vascularity with hyperglycemia in the dark observed in the current study, may leave photoreceptor cells extremely vulnerable to ischemia in disease states such as DR, where the choroidal vessels are already pathologically compromised. Our results suggest that hyperglycemia, especially at night, may exacerbate these metabolic deficits. This hypothesis needs to be verified in future studies through exploring the effects of acute hyperglycemia on CVI and its association with dark adaptometry function in patients with DR.

This study had several limitations. First, it might be considered a limitation that the study did not include different age group or non-myopic population and a lack of flicker stimuli. However, the main purpose of the current study was to investigate the effect of acute hyperglycemia on choroidal structural components and vascularity index while modulating light adaptation in heathy subjects. We used the same group of normal individuals to compare the changes under the two conditions. So, the main results might not be influenced by the sample selection. Nevertheless, because of the limited range of healthy population, the effect of aging (38) or elongation of axial length could not be evaluated. More participants are necessary to explore the effects of aging, elongation of axial length on the response of choroidal structural components and vascularity index. Future studies using a built-in flicker stimulus based on a larger sample size could also provide better insight into the choroidal vascular response to flicker stimulation. Second, this study was also limited by the algorithm used to analyze the choroidal capillaries, which has not yet been investigated. Finally, blood pressure, intraocular pressure, and other physiological variables that could affect the choroidal structures and CVI were not measured in this study. Nevertheless, our study mainly focused on the response of choroidal components, including the lumina and stroma, during dark and light adaptations. We also adjusted for some main factors, including axial length and age, which are known to affect the parameters of choroidal structure components.

In summary, we found that acute hyperglycemia caused a significant decrease in CVI in the dark, which may leave photoreceptors more susceptible to ischemia, especially in disease states where choroid vascularity is compromised. During the transition to ambient light, there was a significant increase in choroidal lumen volume compared with that in the control experiment. These findings suggest that glucose compromises choroidal vascularity. Additional studies are necessary to explore how these findings translate to individuals with diabetes. Analysis of choroidal structural parameters and CVI based on SS-OCT images may be a potentially powerful method to objectively reflect subtle changes in the neurovascular coupling between the choroid and photoreceptor during dark adaptation. Further studies with a longitudinal study design are needed to test choroidal vascular changes in individuals likely to experience a wide range of glucose fluctuations and in patients with diabetes.

## Data availability statement

The raw data supporting the conclusions of this article will be made available by the authors, without undue reservation.

## Ethics statement

The studies involving human participants were reviewed and approved by the Ethics Committee of Wenzhou Medical University. The patients/participants provided their written informed consent to participate in this study.

## Author contributions

ZL, HY, CS, MS, and CW contributed to the study design. ZL and HY contributed to the data collection. ZL, HY, CS, HC and GL contributed to the data analysis and interpretation. ZL, HY, CS, MS, and CW contributed to the manuscript preparation. ZL and HY contributed equally to this work and share first authorship. All authors contributed to the article and approved the submitted version.
