# Supplementary material for: Acute hyperglycemia compromises the responses of choroidal vessels using swept-source optical coherence tomography during dark and light adaptations
Source: Front Endocrinol (Lausanne). 2023 Feb 9;14:1049326. doi: 10.3389/fendo.2023.1049326 (PMC9947340; doi:10.3389/fendo.2023.1049326)
Supplement: Supplementary file 1 [file Table_1.docx]

Supplement Table 1. Mean differences in choroidal parameters change within 0 – 3 mm between glucose and control conditions during light modulation.

|  | **Dark Adaption** | **Light Adaption** | | |
| --- | --- | --- | --- | --- |
|  |  | **30 secs** | **2 min** | **5 min** |
| **Parameter** |  |  |  |  |
| **TCV (mm³)** | -0.02 ± 0.10 | 0.1 ± 0.09 | 0.11 ± 0.10 | 0.19 ± 0.09 |
| **P value** | 0.881 | 0.255 | 0.259 | 0.061 |
| **LV (mm³)** | -0.06 ± 0.06 | 0.07 ± 0.06 | 0.11 ± 0.06 | 0.13 ± 0.06 |
| **P value** | 0.317 | 0.233 | 0.075 | 0.036^*^ |
| **SV (mm³)** | 0.04 ± 0.05 | 0.03 ± 0.04 | 0.003 ± 0.05 | 0.06 ± 0.04 |
| **P value** | 0.391 | 0.421 | 0.947 | 0.183 |
| **CVI (%)** | -0.38 ± 0.13 | 0.1 ± 0.16 | 0.34 ± 0.18 | 0.11 ± 0.14 |
| **P value** | 0.006^*^ | 0.525 | 0.063 | 0.434 |

Mean difference (glucose – control) ± SE.

TCV, total choroidal volume; LV, luminal volume; SV, stromal volume; CVI, choroidal vascularity index.

^*^: P<0.05.
