# Supplementary material for: Acute hyperglycemia compromises the responses of choroidal vessels using swept-source optical coherence tomography during dark and light adaptations
Source: Front Endocrinol (Lausanne). 2023 Feb 9;14:1049326. doi: 10.3389/fendo.2023.1049326 (PMC9947340; doi:10.3389/fendo.2023.1049326)
Supplement: Supplementary file 2 [file Table_2.docx]

Supplement Table 2. Mean differences in choroidal parameters change within 3 – 6 mm between glucose and control conditions during light modulation.

|  | **Dark Adaption** | **Light Adaption** | | |
| --- | --- | --- | --- | --- |
|  |  | **30 secs** | **2 min** | **5 min** |
| **Parameter** |  |  |  |  |
| **TCV (mm³)** | -0.05 ± 0.10 | 0.08 ± 0.08 | 0.13 ± 0.09 | 0.19 ± 0.09 |
| **P value** | 0.611 | 0.319 | 0.162 | 0.049^*^ |
| **LV (mm³)** | -0.07 ± 0.05 | 0.05 ± 0.05 | 0.09 ± 0.05 | 0.14 ± 0.05 |
| **P value** | 0.215 | 0.256 | 0.097 | 0.015^*^ |
| **SV (mm³)** | 0.02 ± 0.05 | 0.03 ± 0.04 | 0.05 ± 0.05 | 0.05 ± 0.05 |
| **P value** | 0.718 | 0.505 | 0.346 | 0.289 |
| **CVI (%)** | -0.33 ± 0.13 | 0.09 ± 0.15 | 0.09 ± 0.14 | 0.22 ± 0.17 |
| **P value** | 0.016^*^ | 0.567 | 0.506 | 0.218 |

Mean difference (glucose – control) ± SE.

TCV, total choroidal volume; LV, luminal volume; SV, stromal volume; CVI, choroidal vascularity index.

^*^: P<0.05.
